# Knowledge and Perception of Facial Candling for Allergic Rhinitis among University Staff and Students

**DOI:** 10.1155/2020/5713134

**Published:** 2020-08-03

**Authors:** Nurul Khaleeda Athiraah Hamdan, Qi Ying Lean, Chin Fen Neoh, Amir Heberd Abdullah, Siong Meng Lim, Kalavathy Ramasamy, Yaser Mohammed Al-Worafi, Khang Wen Goh, Hui Poh Goh, Long Chiau Ming, Pei Lin Lua

**Affiliations:** ^1^Faculty of Pharmacy, Universiti Teknologi MARA, Cawangan Selangor, Kampus Puncak Alam, Bandar Puncak Alam, Selangor, Malaysia; ^2^Faculty of Pharmacy, Universiti Teknologi MARA, Cawangan Pulau Pinang, Bertam Campus, Kepala Batas, Pulau Pinang, Malaysia; ^3^Vector-borne Diseases Research Group (VERDI), Pharmaceutical and Life Sciences CoRe, Universiti Teknologi MARA, Shah Alam, Selangor, Malaysia; ^4^Collaborative Drug Discovery Research (CDDR) Group, Pharmaceutical and Life Sciences Community of Research, Universiti Teknologi MARA, Shah Alam, Selangor, Malaysia; ^5^Department of Environmental Health, Faculty of Health Sciences, Universiti Teknologi MARA, Bertam Campus, Kepala Batas, Pulau Pinang, Malaysia; ^6^College of Pharmacy, University of Science and Technology, Sana'a, Yemen; ^7^College of Pharmacy, University of Science and Technology Faujairah, Fujairah, UAE; ^8^Faculty of Science and Technology, Quest International University Perak, Ipoh, Perak, Malaysia; ^9^PAP Rashidah Sa'adatul Bolkiah Institute of Health Sciences, Universiti Brunei Darussalam, Gadong, Brunei Darussalam; ^10^Faculty of Pharmacy, Universiti Sultan Zainal Abidin, Besut, Terengganu, Malaysia

## Abstract

**Introduction:**

Facial candling is a traditional method used for relieving symptoms of allergic rhinitis (AR). This study aims to investigate the knowledge and perception of facial candling in a sample of staff and students in a public university in Malaysia.

**Methods:**

An online questionnaire survey method was used. Based on sample size calculation, a total of 1,508 UiTM staff and students from ten selected campuses of Universiti Teknologi MARA (UiTM) were invited to participate in this survey. An up-to-date e-mail list of staff in the selected campuses was used as the sampling frame for the study, whereas the students were recruited from the official university student Facebook portal.

**Results:**

A total of 788 respondents participated in this survey, 72.2% of them knew about facial candling, though only 35.4% had tried the treatment. Approximately one-fifth of respondents agreed that facial candling might treat AR. It was found that a higher number of users than nonusers agreed that facial candling was a traditional medicine (78.9% vs 55.0%); could be used on the face and ears (83.5% vs 45.4%); and could be self-administered at home (83.5 vs 45.4%). Interestingly, more than half of them were uncertain about its long-term effects and adverse reactions.

**Conclusion:**

This study confirms the facial candling use among patients with AR although the percentage is low. The patients and general public need to be better informed about the use of facial candling in AR and its associated risks.

## 1. Introduction

Allergic rhinitis (AR) is a symptomatic disorder affecting the upper respiratory system, characterized by nasal inflammation after exposure to external stimuli, such as pollen and house dust [1]. AR affects populations worldwide [2]: 30–40% in the United States [3], 17–29% in Europe [4], and 4–37% in Japan [5]. The classic symptoms of AR include sneezing, itchiness, running nose, and nasal blockage. Ocular symptoms like redness, lacrimation, and conjunctival infection are also commonly experienced [6]. The debilitating symptoms may affect patients' daily activities, work-related productivity, and learning progress in children [7]. They also have a negative impact on social life and self-confidence [8]. Patients with AR have greater risks of sleep apnoea, otitis media, and snoring [9].

It is important to diagnose and manage AR according to the clinical practice guidelines [10]. Nonallergic rhinitis (non-AR) has similar allergy symptoms but a different etiology. While AR reactions are driven by immunoglobulin E, non-AR does not involve the immune system and hence its treatment differs. Therefore, non-AR needs to be differentiated from AR as patients with non-AR are not likely to respond to treatment for AR [11]. The management of AR can be divided into pharmacological and nonpharmacological interventions. Antihistamines, intranasal corticosteroids, and decongestants are most routinely used for providing control and relief for AR [12, 13]. However, some patients still seek complementary alternative medicine (CAM) for their allergies due to the limited success of conventional therapy in relieving the symptoms [14]. Traditional Chinese medicine (TCM), for example, is widely practised in countries like Taiwan, China, Korea, and Japan [15–17]. An epidemiological study in Taiwan found that 57.95% of children were treated with TCM for asthma and AR [16]. Some argued that integrated Chinese-Western treatment is better than western treatment alone [18]. However, the safety of herbal compounds remains underexplored [19].

**Figure 1:**
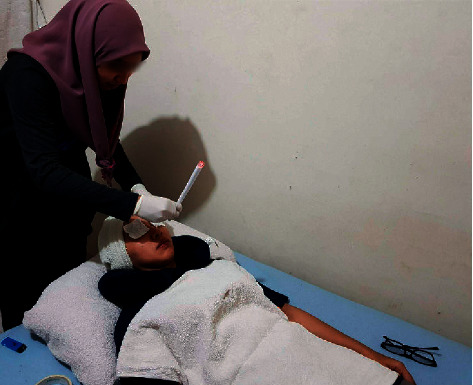
A traditional Malay treatment therapist performs facial candling which involves placing a lit hollow candle which at the other end and the smoke of the burning candle is directed and passed through the face area of the patient.

In Malaysia, there is also an increasing trend in the use and interest in various types of CAM, including aromatherapy, honey consumption, and moxibustion [20–24]. Facial candling, also known as *lilin resdung* in Malay, has become a popular traditional Malay treatment for AR [25]. According to the definition of the American Academy of Audiology, an ear candle is made from cloth soaked in beeswax or paraffin blended from selective traditional tropical herbs [26]. Like ear candles, facial candles are hollow cones with wax. In some local settings, facial candles are made from organic herbal materials such as Indian traditional medicines, pure honey, cinnamon, and wool. Various types of facial candles such as beeswax and aromatherapy wax are available for purchase, either at local Malay herbal treatment centres/shops or online. Some also create facial candles by rubbing candles and herbs onto a clean sheet of paper and rolling it into a tube. Facial candling can be performed by beauticians, specialist therapists, or even the patients themselves [27]. During the treatment, the facial candle is lighted at one end, and smoke is produced at the other end of the burning candle. The smoke is directed to pass through the certain facial areas, especially areas around the nose (Figure 1).

The use of facial candling originates from the myths and beliefs surrounding traditional Malay medicinal practices. Most patients believe that facial candling could cure or reduce the symptoms of AR by killing the germs or expelling “worm” from the sinus cavities [25]. The treatment claims to help in releasing “toxins” from the body and to alleviate stress as it promotes relaxation. By doing so, it is supposed to relieve headache, remove ear and throat infections, and improve hearing. Besides, facial candling is also said to be able to improve blood circulation in the body [25].

The benefits of facial candling treatment are still inconclusive since no clinical studies have been conducted on its effectiveness and safety. Nevertheless, the practice is well accepted by Malaysians, especially the ethnic Malays [6]. The use of facial candles is widespread in the Malay community, especially in places where there is word-of-mouth publicity from patients who have benefited the treatment. Therefore, studies in regard to the perception of the community about facial candling are important in understanding the current influence of this practice. We aim to evaluate the understanding of AR, knowledge, and perception of facial candling and the potential factors associated with the use of facial candling in AR among university staff and students. It is crucial to investigate the use and acceptance of facial candling among this group of the population, who have good access to healthcare information, as this served as a first step to investigate its usefulness and safety in managing AR.

## 2. Methods

### 2.1. Study Design

An online survey was conducted over a period of three months to explore the perception about facial candling and its use for AR among university staff and students at Universiti Teknologi MARA (UiTM). UiTM is a local public university, which has 34 campuses across Malaysia offering a wide variety of programmes from diploma to postgraduate levels. This study was approved by the Research Ethics Committee of UiTM (600-RMI-5/1/6).

### 2.2. Participants and Sampling Method

There were approximately 17,000 staff and 16,0000 students across the 34 UiTM campuses. Out of the 34 campuses, ten campuses were included based on geographical locations. A total of 1,508 respondents were invited to participate in this survey. These include 160 respondents each from UiTM campuses located in Shah Alam, Dungun, Machang, Puncak Alam, Bertam, Tanah Kuala Muda, Bandaraya Melaka, and Pasir Gudang as well as 114 respondents each from Tawau and Samarahan.

The sampling method used by Magliano et al. [28] was adopted. Firstly, the sample population was proportionately grouped based on the number of university students and staff in the ten selected campuses. Secondly, the grouped sample was also chosen to ensure proportional representation of the university. The shortlisted staff were invited through an invitation e-mail sent to the UiTM e-mail addresses. An up-to-date e-mail list of staff in the selected ten campuses were obtained from the Human Resource Department of the university, whereas the students were recruited from the official university student Facebook portal which was linked to the students at the selected campuses.

For a 95% confidence level based on the Raosoft's sample size calculator, the minimum number of respondents required for this study was 377. By taking into consideration the usual low response rate for online surveys, we targeted 1,508 potential respondents, which was four-fold of the minimum number required. Those who could not understand Malay language were excluded from this study. Informed consent was acquired before respondents started answering the survey online. Meanwhile, formal consent was obtained from the practitioner and the patient, during a treatment session, for publishing an image of facial candling (Figure 1). Their participation in the study was anonymous, with no personal details collected.

### 2.3. Development of the Questionnaire

A self-administering questionnaire was developed in Malay language, based on literature on CAM [29–31] and AR [2, 13, 17]. The questionnaire consisted of three section: the demographic of the participants (Section A: 6 questions); knowledge of AR and facial candling (Section B: 24 questions); and for facial candling users only, user experience, and perceived factors that influence their opting for facial candling (Section C: 18 questions). For Section B, yes/no questions were used. For Section C, a Likert scale from agree, not sure, to disagree was used to reflect the level of understanding and influence of perceived factors in the use of facial candling in treating AR.

The questionnaire was first checked by two senior lecturers from the Faculty of Pharmacy for its face and content validity. Their feedback was used to improve the questionnaire. A pilot study of 15 staff and students was then performed to check the reliability and validity of the questionnaire. Data collected from the pilot study were not included in the final study. The internal reliability of the questionnaire was assessed by Cronbach's alpha coefficient, which reported a value of 0.83.

### 2.4. Data Analysis

The collected data were analysed using the Statistical Package for Social Sciences, version 20 (SPSS Inc., Chicago, IL, USA). Descriptive statistics were used to report demographic characteristics. A chi-squared test was used to test the association of facial candling use with demographic variables. For all statistical analysis, the result was considered significant if its *p* value was less than 0.05.

## 3. Results

### 3.1. Respondents' Characteristics

A total of 788 participants responded to the survey (Table 1). The response rate, therefore, was 52.3%. More than half of the respondents were female (64.7%). The majority of the respondents were Malays (89.7%), followed by aboriginal ethnicities from Sabah and Sarawak (5.7%), Chinese (3.8%), and Indian (0.8%). Most respondents were in the age groups of 20–29 (33.5%) or 30–39 (42.8%). Most were married (70.4%) and had received tertiary education (89.0%). Out of the 788 respondents, a significant minority, 35.4% (*n* = 279), had received facial candling. Facial candling use was found to be higher in females (65.6%) than in males. Our findings showed no significant correlation between the use of facial candling and gender (*p*=0.349) as well as education level (*p*=0.258). The age group (*p*=0.011) and ethnicity (*p*=0.004) were significantly associated with facial candling use. The majority of facial candling users were below 40 years old (76.7%) and were Malays (94.6%).

### 3.2. Respondents' Knowledge of AR

We first determined patients' knowledge on AR (Table 2). Of 788 participants, 55.8% (*n* = 440) claimed that they suffered from AR. The majority of respondents (89.5%, *n* = 705) agreed that AR was an inflammation of the sinus and usually due to blockage. A total of 72.2% (*n* = 569) of the participants reported that AR had clearly disturbed their daily activities. The majority (90.5%, *n* = 713) of respondents believed that AR was not contagious. Most of the respondents (71.8%, *n* = 566) also thought that the condition was not easy to cure. More nonusers (67.8%) than facial candling users (39.4%) believed that AR was not a genetic disease. Although most respondents (69.7%, *n* = 549) presumed that medicines for treating AR were generally available, a higher percentage of facial candling users (83.9%, *n* = 234) than nonusers (69.7%, *n* = 355) believed that most treatments were sourced from traditional medicine.

### 3.3. Respondents' Knowledge of Facial Candling Treatment in AR

We then sought to understand the knowledge of facial candling among respondents (Table 3). A total of 70.8% (*n* = 558) of respondents claimed that they knew about facial candling. While all facial candling users claimed they knew about facial candling, only approximately half (54.8%) of nonusers did so. Overall, higher percentage of users (30.5%) than nonusers (16.1%) agreed that facial candling could treat AR. Up to one-fifth of users agreed that facial candling was their first choice to treat AR, whereas only 7.1% of nonusers thought so. The majority of the users (78.9%–88.5%), compared with approximately half (45.0%–55.0%) of nonusers, agreed that facial candling involved the use of traditional herbal medicine, and it could be used for the face and ears. It was found that a higher percentage of users than nonusers were aware that facial candling could be carried out by users at home (83.5% vs 45.4%); in a salon (73.8% vs 31.2%); could be done at any time (79.6% vs 43.6%); and used by everyone (53.8% vs 35.4%). Similarly, higher proportion of users (48.7%) than nonusers (36.5%) believed that there was a “worm” coming out from the nose during the treatment. Interestingly, more nonusers (37.5%) compared to users (28.7%) expected that facial candling would be effective in reducing itchiness of the face, ears, and eyes. In terms of side effects, more than half of respondents (58.4%, *n* = 460), especially nonusers (81.7%, *n* = 416), were uncertain whether facial candling might irritate the eyes. Also, a total of 82.9% of nonusers did not know that asthma patients should not receive facial candling. Moreover, it is important to note that a minority of both users (29.0%) and nonusers (13.8%) believed that a frequent use of facial candling could reduce one's allergy to dust.

### 3.4. Users' Experience and Perceived Influences of Facial Candling Use

Table 4 summarizes the facial candling users' experience. Users received facial candling from traditional medical centres (38.1%), at salons (47.4%), or at home (14.4%). Among the different sources from which the users learned about facial candling, family and friends (63.4%) were found to be most influential source. Among the users, facial candling was usually sought upon experiencing certain symptoms, particularly itchiness at the face and nose (42.7%), sneezing (20.6%), and runny nose (23.7%). The most common side effects of facial candling reported by users include redness (36.8%) and itchiness (32.1%) at the facial area after the treatment. Meanwhile, 58.2% of the users had tried this treatment up to three times while 8.8% had received the treatment more than 10 times. The cost of treatment was mostly below MYR40 (78.0%) (MYR 1 is equivalent to USD 0.27).

We then asked users questions pertaining to factors that led to the use of facial candling (Table 5). Users reported that user-friendliness (67.0%), relatively affordable price (55.5%), and accessible treatment sites (57.3%) were the main reasons of facial candling. Approximately one-fifth of the users (23.7%) received the treatment because of their trust in traditional Malay medicine. On the other hand, 38.7% of users sought for alternative medicine only when modern treatment was ineffective. Up to 40.1% of the users believed that facial candling could improve their general health. A minority (12.2%) presumed that AR was an uncomplicated condition that did not require medical attention. Also, a small percentage of the users (15.1%) believed that they did not need other medicines since facial candling had a sustainable effect (12.2%) and was more effective than modern treatment (12.9%). Nonetheless, the majority (58.4%) were uncertain whether its use was associated with any side effects.

## 4. Discussion

This study is the first instrumental baseline study aimed at describing the knowledge and perception of facial candling for AR in a Malaysian public university that has a Malay majority in its staff and students. Although the majority of respondents knew about facial candling, only 35.4% had actually tried it. The percentage of AR patients opting for facial candling was lower than the percentage of patients using other CAM in different medical conditions in Malaysia [31–33]. For example, up to 46.1% of patients used CAM for treating cancers; 63-64% used it for chronic diseases such as inflammatory disorders, diabetes mellitus, and cardiovascular diseases [16, 32, 34]. Due to unmet pharmacological needs in relieving AR, patients commonly might seek out alternatives including self-medication and CAM. Because of a wide selection of other types of CAM for AR, patients might be less likely to opt for facial candling. Their choice might also be due to a better confidence in using dosage forms which have a higher visibility of the herbal ingredients, such as the oral or nasal herbal preparations, as most respondents believed that medicines for AR should be made from traditional medicines.

In this study, most facial candling users were ethnic Malays. This is because Malay is the largest ethnic group in Malaysia, constituting almost 62% of the total population. Likewise, more than 90% of the university staff and students in UiTM were ethnic Malays, who had contributed to a large percentage of the respondents in this study. Furthermore, facial candling potentially has a higher popularity among the Malay community as it is one of the Malay traditional medicines. It is widely accepted by the Malay patients, despite the majority may not be interested in learning the scientific knowledge of the medicinal practices [35]. Meanwhile, Ayurvedic medicine and Traditional Chinese Medicine (TCM) are more popular in Indian and Chinese ethnic groups, respectively [36, 37].

Approximately the bare majority of nonusers were aware of the availability of facial candling as a treatment for AR. This is in contrast to the common assumption about university staff and students being knowledgeable about the practice of CAM, as facial candling is widely practised in beauty salons. As in previous reports [32, 34, 38], family and friends were the most influential to patients in accepting CAM for treating their conditions. Similarly, in this study, family and friends were identified as the most common source of reference and influence for the use of facial candling.

Unlike TCM, the use of facial candling is still in its infancy. Its practices, including types of herbal products and procedures at traditional treatment centres, are not subjected to quality control and medical regulation [6]. The centres are commonly run by practitioners who are not certified or have not received formal education in CAM practices. Facial candling is only favoured because of the claims and advertisements of the treatment centres on its various beneficial functions [14]. Besides, most of respondents who used facial candling agreed that they learned about facial candling from their social network. This might be due to the fact that a highly connected social network among the Malay community speeds up the spread of any health-related information [39].

Besides, the selection of facial candling might be due to patients' personal belief and desperate for a cure. The users' higher trust in the usefulness of facial candling may be because they had experienced the treatment and symptom relief. This is reflective of the results on respondents' first-choice treatment for AR, while higher percentage of users compared to nonusers preferred facial candling. Facial candling is also generally well received in the community as it is user-friendly, convenient, easy to use, and affordable. This was in line with other findings as the main reasons for CAM use were the affordability and convenience [17]. In contrast, results for nonusers may indirectly imply a lack of efficacy or even negative experience of facial candling either in their cycle of friends or relatives. Nonetheless, the use of CAM including facial candling among Malaysians should not be negligible even though there are relatively small number of users who believe in its potential [40].

Although some had a positive perception towards facial candling, most respondents were unclear about the effectiveness and side effects of facial candling. This is aligned with findings in other studies as most patients were having very little or no knowledge of CAM [41]. In this study, adverse effects associated with facial candling were reported. This warrants a concern as the smoke from the burning candle may become an allergen especially to patients with asthma. There is also a risk of burn or irritation resulting from the flame and ashes. In fact, complications such as burns from hot wax, ear canal blockage from candle wax, and eardrum perforation have been well documented for ear candling [27]. The lack of knowledge regarding the harmful effects of this conventional therapy among the users may partially be due to an inadequate information available about traditional Malay medicines including facial candling. Also, there were some false beliefs regarding the unnecessity of modern medicine when receiving facial candling. Chronic inflammatory conditions without proper medical management could pose a considerable risk to the users if facial candling appears to be a popular replacement therapy for patients with AR. Therefore, during any clinic or pharmacy visits, it is also important for healthcare professionals to take a CAM history during patient visits at the clinic or pharmacy. This would help to increase knowledge of CAM use and to prevent associated adverse reactions [42]. It must be noted that a methicillin-resistant *Staphylococcus aureus* (MRSA) nasal carriage prevalence study conducted at the state of Terengganu revealed that 32% (*n* = 119) of the 370 volunteers from a public university carried *S. aureus* with 18 of the isolates being MRSA. The findings are rather alarming because MRSA with staphylococcal cassette chromosome mec (SCCmec) of hospital-associated features was found in this prevalence study [43].

Facial candling remains one of the popular traditional treatments in the community, though without good evidence for its safety and effectiveness. Recently, there was a study proposal to evaluate the immediate effect of facial candling on inflammatory mediators, symptom severity, and patients' quality of life [6]. It is important to elucidate the underlying mechanism of herbal medicines in facial candles. The perceived effects of facial candling on AR also could not predict the clinical outcomes and patients' quality of life. Facial candling may only be recommended as an alternative therapy for AR if efforts are made in ensuring its safety and effectiveness, including having a good quality control in the manufacturing of facial candles, standard procedures in its administration, and certification for its practitioners.

## 5. Strengths and Limitations of Study

This was an online questionnaire survey, so respondents were invited to participate through emails and/or advertisements on social media platforms. The majority of the respondents received higher education from a public university. Although further studies are required to apply the findings to the Malaysian populations, this study provides some data on the perception of the Malaysian public in particular the Malay population, who are the most common users of facial candling. Because of the use of a self-administered questionnaire, the survey was unable to distinguish actual AR from provisional or differential diagnosis of AR or to identify types and severity of AR. Participants' reliance on own memories in respect to facial candling use might result in inaccuracy. The present study also did not evaluate the health outcomes of facial candling.

## 6. Conclusion

This study investigates the knowledge of facial candling use in AR. Only minority tried facial candling although the majority of respondents knew about facial candling. Overall, patients' feedback did not support the effectiveness of facial candling for the treatment of AR. Nonetheless, facial candling is a traditional Malay medicine deliberately practiced in the Malaysian community setting due to belief and hope. Thus, there is a need for patient education regarding its use and potential side effects or complications. Additional research is required to assess the practice of facial candling before it is accepted as a CAM to treat AR. Given the high prevalence of allergic conditions and associated costs of CAM treatments, the evidence for the safety and effectiveness of facial candling is needed to establish appropriate guidelines for its use as one of the CAM modalities.

## Figures and Tables

**Table 1:** Demographic characteristics of study population.

Variables	*n* (%)			
	Facial candling users *n* = 279	Nonusers *n* = 509	Total (*n* = 788)	*p* value
Gender				
Male	96 (34.4)	182 (35.8)	278 (35.3)	0.359
Female	183 (65.6)	327 (64.2)	510 (64.7)	

Age (years)				
10–19	1 (0.4)	1 (0.2)	2 (0.3)	0.011
20–29	81 (29.0)	183 (36.0)	264 (33.5)	
30–39	132 (47.3)	205 (40.3)	337 (42.8)	
40–49	49 (17.6)	50 (9.8)	99 (12.6)	
≥50	16 (5.7)	70 (13.8)	86 (10.9)	

Ethnicity				
Malay	264 (94.6)	443 (87.0)	707 (89.7)	0.004
Chinese	3 (1.1)	27 (5.3)	30 (3.8)	
Indian	0 (0)	6 (1.2)	6 (0.8)	
Others	12 (4.3)	33 (6.5)	45 (5.7)	

Marital status				
Single	72 (25.8)	155 (30.4)	227 (28.8)	0.574
Married	205 (73.5)	350 (68.8)	555 (70.4)	
Divorce	2 (0.7)	4 (0.8)	6 (0.8)	

Education level				
Secondary school	9 (3.2)	78 (15.3)	87 (11.0)	0.068
University/college	270 (96.8)	431 (84.7)	701 (89.0)	

Occupation				
Government servant	254 (91.0)	445 (87.4)	699 (88.7)	0.286
Student	25 (9.0)	64 (12.6)	89 (11.3)	

**Table 2:** Patients' knowledge about AR (*n* = 788).

Variables	User *n* = 279		Nonuser *n* = 509		
	Yes	No	Yes	No	Undisclosed
1. AR is also known as inflammation of the sinus	277 (99.3)	2(0.7)	428 (84.1)	79 (15.5)	2 (0.4)
2. Usually AR happened due to the blockage of the sinus as well as the nose	271 (97.1)	8 (2.9)	440 (86.4)	62 (12.2)	7 (1.4)
3. I know the symptoms of AR	278 (99.6)	1 (0.4)	432 (84.9)	54 (10.6)	23 (4.5)
4. AR is easy to be cured	58 (20.8)	221 (79.2)	157 (30.8)	345 (67.8)	7 (1.4)
5. AR is a genetic disease.	169 (60.6)	110 (39.4)	145 (28.5)	345 (67.8)	19 (3.7)
6. AR is contagious.	22 (7.9)	257 (92.1)	42 (8.3)	456 (89.6)	11 (2.2)
7. Does AR decrease daily routine of individual?	217 (77.7)	62 (22.3)	352 (69.2)	137 (26.9)	20 (3.9)
8. It is easier to get medicines for AR	216 (77.4)	63 (22.6)	333 (65.4)	148 (29.1)	28 (5.5)
9. Generally, treatment of AR comes from traditional medicine	234 (83.9)	45 (16.1)	355 (69.7)	143 (28.1)	11 (2.2)
10. I have AR	234 (83.9)	45 (16.1)	206 (40.5)	296 (58.2)	7 (1.4)

**Table 3:** Patients' knowledge of facial candling for the treatment of AR.

Variables	User *n* = 279			Nonuser *n* = 509			
	Agreed	Disagreed	Not sure	Agreed	Disagreed	Not sure	Undisclosed
1. I know about facial candling	279 (100)	0 (0)	0 (0)	279 (54.8)	201 (39.5)	28 (5.5)	1(0.2)
2. Facial candling treatment can treat AR	85 (30.5)	32 (11.5)	162 (58.1)	82 (16.1)	38 (7.5)	388 (76.2)	1 (0.2)
3. Facial candling treatment can be used at the face and ear	247 (88.5)	2 (0.7)	30 (10.8)	229 (45.0)	16 (3.1)	263 (51.7)	1 (0.2)
4. Facial candling treatment can be used anytime	222 (79.6)	10 (3.6)	47 (16.8)	222 (43.6)	21 (4.1)	270 (53.0)	1 (0.2)
5. Facial candling treatment involves the use of herbal traditional medicines	220 (78.9)	8 (2.9)	51 (18.3)	280 (55.0)	17 (3.3)	211 (41.5)	1 (0.2)
6. Facial candling treatment can be used for everyone	150 (53.8)	30 (10.8)	99 (35.5)	180 (35.4)	45 (8.8)	283 (55.6)	1 (0.2)
7. Usually, facial candling treatment in salon will be finished with massage around the face	206 (73.8)	11 (3.9)	62 (22.2)	159 (31.2)	10 (2.0)	339 (66.6)	1 (0.2)
8. Facial candling treatment can be done ourselves at home	233 (83.5)	10 (3.6)	36 (12.9)	231 (45.4)	27 (5.3)	250 (49.1)	1 (0.2)
9. Facial candling treatment is the first choice to treat AR	60 (21.5)	74 (26.5)	145 (52.0)	36 (7.1)	110 (21.6)	362 (71.1)	1 (0.2)
10. It has been said that, after facial candling treatment, there would be “worm” coming out from nose	136 (48.7)	52 (18.6)	91 (32.6)	186 (36.5)	35 (6.9)	287 (56.4)	1 (0.2)
11. Facial candling treatment can decrease the itchiness at the face, eyes, and ears	80 (28.7)	18 (6.5)	181 (64.9)	191 (37.5)	13 (2.6)	304 (59.7)	1 (0.2)
12. Asthma patients are not recommended to use facial candling treatment	80 (28.7)	18 (6.5)	181 (64.9)	76 (14.9)	10 (2.0)	422 (82.9)	1 (0.2)
13. Facial candling treatment can irritate the eyes	160 (57.3)	75 (26.9)	44 (15.8)	70 (13.8)	22 (4.3)	416 (81.7)	1 (0.2)
14. Allergy to dust can be decreased with frequent use of facial candling treatment	81 (29.0)	31 (11.1)	167 (59.9)	70 (13.8)	31 (6.1)	407 (80.0)	1 (0.2)

**Table 4:** Facial candling users' experience (*n* = 279).

Variables	Total^*∗*^, *n* (%)
Places of facial candling	
Salons	128 (47.4)
Traditional medicine centres	103 (38.1)
Home treatment	39 (14.4)

Sources of information about facial candling	
Friends/family	220 (63.4)
Newspaper/magazine	47 (13.5)
Radio/television	14 (4.0)
Social networks	56 (16.1)
Others	10 (2.9)

Symptoms leading to facial candling	
Sneezing	130 (20.6)
Runny nose	149 (23.7)
Inflammation of the nose	54 (8.5)
Itchy at face and nose	269 (42.7)
Others	28 (4.4)

Cost per treatment (MYR)	
<10	56 (24.7)
10–19	47 (20.7)
20–29	56 (24.7)
30–39	18 (7.9)
40–49	30 (13.2)
≥50	20 (8.8)

Number of times of treatment	
≤3	152 (58.2)
4–9	86 (33.0)
10–19	14 (5.4)
20–29	6 (2.3)
>30	3 (1.1)

Side effects encountered after facial candling	
Itchiness at face	89 (32.1)
Sneezing frequently	53 (19.1)
Redness at face area	102 (36.8)
Acne	30 (10.8)
Others	3 (1.1)

^*∗*^Respondents could have more than one response.

**Table 5:** Perceived factors that influence the use of facial candling (*n* = 279).

Variables	Agreed	Disagreed	Not sure
	*n* (%)		
1. I do not prefer modern treatment because I believe more to traditional medicine	66 (23.7)	136 (48.7)	77 (27.6)
2. I will use facial candling treatment if modern treatment is not working	108 (38.7)	103 (36.9)	68 (24.4)
3. Price of facial candling treatment is cheaper and reasonable	155 (55.5)	38 (13.6)	86 (30.8)
4. Facial candling treatment is easier to use	187 (67.0)	46 (16.5)	46 (16.5)
5. Facial candling treatment can improve my health	112 (40.1)	55 (19.7)	112 (40.1)
6. Facial candling treatment has no side effect	86 (30.8)	30 (10.8)	163 (58.4)
7. The effect of facial candling treatment is long term	34 (12.2)	87 (31.2)	158 (56.6)
8. Facial candling treatment is much better than modern treatment	36 (12.9)	63 (22.6)	180(64.5)
9. After undergoing facial candling, I did not need other medicines	42 (15.1)	156 (55.9)	81 (29.0)
10. AR is a simple disease and does not need doctor's advice	34 (12.2)	174 (62.4)	71 (25.4)
11. Facial candling treatment is easier to reach and near to my home	160 (57.3)	52(18.6)	67 (24.0)
12. Most of my family member used facial candling to treat AR	55 (19.7)	148 (53.0)	77 (27.6)

## Data Availability

The data used to support the findings of this study are included within the article.

## Abbreviations

AR: Allergic rhinitis
CAM: Complementary alternative medicine
UiTM: Universiti Teknologi MARA
MYR: Malaysian currency ringgit.
